# Low‐intensity pulsed ultrasound ameliorates depression‐like behaviors in a rat model of chronic unpredictable stress

**DOI:** 10.1111/cns.13463

**Published:** 2020-10-28

**Authors:** Jinniu Zhang, Hui Zhou, Jian Yang, Jun Jia, Lili Niu, Zuoli Sun, Dandan Shi, Long Meng, Weibao Qiu, Xiaomin Wang, Hairong Zheng, Gang Wang

**Affiliations:** ^1^ The National Clinical Research Center for Mental Disorders & Beijing Key Laboratory of Mental Disorders Beijing Anding Hospital Capital Medical University Beijing China; ^2^ Paul C. Lauterbur Research Center for Biomedical Imaging Institute of Biomedical and Health Engineering Shenzhen Institutes of Advanced Technology Chinese Academy of Sciences Shenzhen China; ^3^ Key Laboratory for the Neurodegenerative Disorders of the Chinese Ministry of Education Department of Physiology Capital Medical University Beijing China; ^4^ Advanced Innovation Center for Human Brain Protection Capital Medical University Beijing China

**Keywords:** brain‐derived neurotrophic factor, depression, low‐intensity pulsed ultrasound, mammalian target of rapamycin complex 1, ventromedial prefrontal cortex

## Abstract

**Introduction:**

There is an unmet need for better nonpharmaceutical treatments for depression. Low‐intensity pulsed ultrasound (LIPUS) is a novel type of neuromodulation that could be helpful for depressed patients.

**Objective:**

The goal of this study was to investigate the feasibility and potential mechanisms of LIPUS in the treatment of depression.

**Methods:**

Chronic unpredictable stress (CUS) was used to generate rats with depression‐like features that were treated with four weeks of LIPUS stimulation of the ventromedial prefrontal cortex. Depression‐like behaviors were assessed with the sucrose preference, forced swim, and open field tests. BDNF/mTORC1 signaling was examined by Western blot to investigate this potential molecular mechanism. The safety of LIPUS was evaluated using hematoxylin‐eosin and Nissl staining.

**Results:**

Four weeks of LIPUS stimulation significantly increased sucrose preference and reduced forced swim immobility time in CUS rats. LIPUS also partially reversed the molecular effects of CUS that included decreased levels of BDNF, phosphorylated tyrosine receptor kinase B (TrkB), extracellular signal‐regulated kinase (ERK), mammalian target of rapamycin complex 1 (mTORC1), and S6 kinase (S6K). Moreover, histological staining revealed no gross tissue damage.

**Conclusions:**

Chronic LIPUS stimulation can effectively and safely improve depression‐like behaviors in CUS rats. The underlying mechanisms may be related to enhancement of BDNF/ERK/mTORC1 signaling pathways in the prefrontal cortex (PFC). LIPUS is a promising noninvasive neuromodulation tool that merits further study as a potential treatment for depression.

## Graphical abstract

Four weeks repetitive transcranial low intensity pulsed ultrasound stimulation ameliorates depression‐like behaviors in a depression model of chronic unpredictable stress.

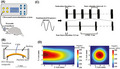

## 1 INTRODUCTION

Major depressive disorder (MDD) is one of the most common psychiatric illnesses, and it causes substantial disability and mortality.^1^ Antidepressants are effective for 60%‐70% of MDD patients,^2^ but some patients who do not respond, and long‐term treatment with antidepressants may cause distressing side effects.^3^ Depression is associated with brain dysfunction resulting from disturbed neural circuits.^4^ Increasing evidence suggests that nonpharmaceutical neuromodulation techniques are capable of regulating brain circuit activity and thus are worthwhile to investigate as a potential therapy for depression.

Ultrasound is used in a wide range of diagnostic imaging applications. Therapeutic ultrasound at a higher power can be used to induce damage of focused lesions. This decade, therapeutic ultrasound techniques are being developed to induce neuromodulation by activating or suppressing neuronal circuits. Low‐intensity pulsed ultrasound (LIPUS) has been of interest as a neuromodulation tool. LIPUS is similar to high‐intensity focused ultrasound (HIFU) in being able to penetrate the intact skull to target subcortical structures with high spatial resolution. However, HIFU ablates brain tissue through thermal effects,^5^, ^6^, ^7^ while LIPUS does not damage the brain.^8^, ^9^, ^10^, ^11^, ^12^ Depending on the intensity, frequency, and exposure time, LIPUS can either activate or suppress neuronal activity partially through producing mechanical vibration of the cellular membrane.^13^, ^14^ The underlying mechanisms are thought to be mainly mediated by various mechanosensitive ion channels,^12^, ^15^, ^16^ such as two‐pore domain potassium channels (TREK‐1, TREK‐2, TRAAK),^17^ transient receptor potential (TRP)‐4,^18^ Piezo1^19^ and MscL.^16^

Animal studies support the idea that LIPUS may be useful in treating brain disorders. LIPUS can modify behavior,^20^ and exert therapeutic effects in animal models of several neurological disorders, including ischemic brain injury,^21^, ^22^ Parkinson's disease,^23^ Alzheimer's disease,^24^, ^25^, ^26^ epilepsy,^27^, ^28^ and essential tremor.^29^ The ultrasound devices used in previous researches are usually huge and difficult to operate.^12^, ^22^, ^30^ Instead, we used a portable ultrasound system that has been developed independently.^31^ This portable ultrasound system is much more convenient for researchers to achieve ultrasonic neurostimulation. Compared with previous studies,^12^, ^22^ we applied LIPUS to awake and free‐moving animals rather than anesthetized ones. This not only avoids the possible effects of anesthesia on the behavior, but also allows us to observe animal behavior during the stimulation. This way seems more suitable for future application in clinic. A recent study also reported that LIPUS alleviated depression‐like behavior in rats subjected to 48‐h restraint stress while upregulating expression of brain‐derived neurotrophic factor (BDNF) in the hippocampus.^30^ However, acute restraint is not the best way to simulate longer‐term psychosocial stressors that can contribute to depression. We used the chronic unpredictable stress (CUS) model because it relates well with the time course of the condition in the clinic.^32^ In addition, the mechanisms by which LIPUS may modulate depression‐related behaviors need further investigation. The mTORC1 pathway is involved in neuronal differentiation, neuron migration, synaptic plasticity,^33^ and the effects of antidepressant medications.^34^, ^35^ However, the mechanism of its antidepressant effect in LIPUS stimulation is still unclear.

Therefore, the present study aims to explore the feasibility and potential mechanisms of LIPUS in the treatment of depression using the well‐known chronic unpredictable stress (CUS) model in the rat.^36^ First, we examined the effects of LIPUS applied to the ventromedial prefrontal cortex (vmPFC), on depression‐like behaviors in CUS rats. The vmPFC was chosen because it plays an important role in emotion regulation.^37^ Of the locations at which deep brain stimulation (DBS) is effective in treating depression, the vmPFC is the most readily targeted by LIPUS.^38^, ^39^ Other deep brain structures are covered with dense white matter tracts which may absorb or scatter ultrasound waves.^12^ The second aim is to investigate the role of the mammalian target of rapamycin complex 1 (mTORC1) signaling pathway in the effects of LIPUS. The third aim is a preliminary exploration of the safety of LIPUS.

## 2 MATERIALS AND METHODS

### 2.1 Animals and CUS procedures

The protocol was approved by the Animal Use and Care Committee at Capital Medical University (AEEI‐2018‐003). All efforts were made to minimize animal suffering.

Thirty 6‐ to 8‐week‐old male Sprague‐Dawley (SD) rats (150‐180 g) provided by Beijing Vital River Laboratory Animal Technology Co., Ltd. were housed at a controlled ambient temperature of 23 ± 2°C with 50 ± 10% relative humidity under a 12‐h light‐dark cycle. Before the experiment, all the rats were adaptively fed for one week. Food and water were given ad libitum apart from the periods of stress and behavioral measurements. Before CUS, animals were randomly (stratified random) divided into the blank control group (n = 8) and CUS group (n = 22), according to baseline sucrose preference indices (SPI). Stressed and nonstressed animals were housed in different rooms. The rats of control group were housed in pairs in home cages with the floor covered with corncob and had free access to food and water except before the SPI test. Rats in the CUS group were housed in individual cages and subjected to one or two of fourteen stressors per day on a weekly schedule for six consecutive weeks. These included three 1‐minute periods of 36 V electric foot shock, one 5‐minutes period of swimming in cold water (4°C), two 5‐minutes periods of swimming in hot water (45°C), five 2‐hour periods of immobilization using rat fixator that made of polymethyl methacrylate, six 24‐hour periods of water deprivation, two 24‐hour periods of food deprivation, three 1‐minute periods of tail clamp, three 24‐hour periods of wet bedding, one 24‐hour period of no padding, three 24‐hour periods of soiled cage, four 24‐hour periods with cage titling at 45°, six 2‐hour periods of 85 dB noise, three 24‐hour periods with continuous illumination and two 2‐hour periods of stroboscopic illumination. All the rats in stressed group were subjected to the same one or two stressors on the same day. At the end of 6‐week CUS procedures, 16 depression rat models were established successfully. And the 16 models were redistributed into CUS + sham LIPUS and CUS + LIPUS groups according to the SPI.

### 2.2 Parameters screening and testing

Normal 10‐ to 12‐week‐old male SD rats (400‐500 g) were used to screen suitable stimulation parameters. They were divided into two groups: control and LIPUS groups (n = 6‐8 rats per group). We screened three different stimulation parameters. In the LIPUS group, rats were treated with single LIPUS stimulation with three different parameters in batches for 30 minutes, and behavioral test was carried out 24 hours later. In the control group, the rats underwent the same procedures, but the power was turned off. An ideal experimental design only needs a control group. However, the operation of LIPUS stimulation is very time‐consuming and each rat needs 45 minutes to operate. Meanwhile, we have only two home‐made wearable transducers. Under these conditions, in order to ensure adequate sample size in each group, we could at most stimulate two groups (including the control group, which underwent the same procedures without ultrasound stimulation) of rats every day. Therefore, considering the feasibility of practical operation, we tested the effects of different parameters of LIPUS stimulation separately.

Normal and CUS rats were used to examine the effects of chronic LIPUS stimulation. They were control, CUS + sham LIPUS and CUS + LIPUS groups (n = 8 rats per group). After 6‐week CUS exposure, each rat was fixed with an ultrasound collimator on the skull and was allowed 1 week to recover from surgery. Then, ultrasound stimulation was applied to awake and freely moving rats in the CUS + LIPUS group, for twenty minutes each day for four consecutive weeks. Rats in the control and CUS + sham LIPUS groups underwent the same procedures, but the power was turned off. The CUS procedures were continued during the LIPUS application.

**Figure 1:**
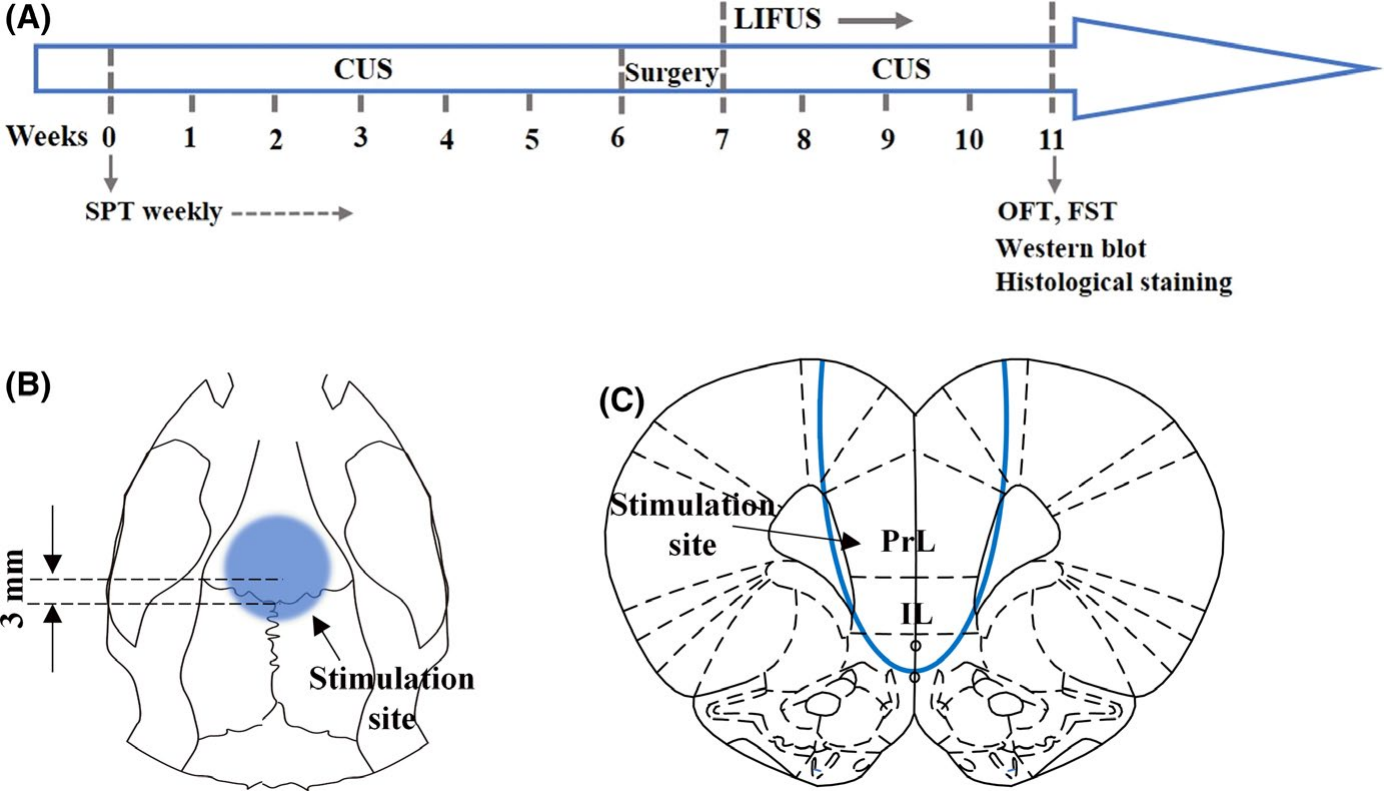
Overview of the experimental procedures. A, The timeline of the experiments. After 1 week of adaptation, the control group were fed normally in pairs. Rats in the CUS group were housed in individual cages and subjected to one or two of fourteen randomly selected different stressors per day for six consecutive weeks. After completion of the CUS exposure, each rat was fixed with an ultrasound collimator on the skull and was allowed 1 week to recover from surgery. Normal control rats had holes drilled into the skull, but the collimator was not affixed. Then, ultrasound stimulation was applied to awake and freely moving rats in the CUS + LIPUS group, for twenty minutes each day for four consecutive weeks. Rats in the control and CUS + sham LIPUS groups underwent the same procedures, but the power was turned off. Behavioral tests were conducted immediately after LIPUS. After that, rats were sacrificed, and their brains were removed for morphological and biochemical experiments. (B, C) Anatomical schematic of the stimulation area. A custom‐designed acoustic collimator was fixed on the skull covering the vmPFC (The center of the circle is anteroposterior + 3.0 mm; medial‐lateral 0 mm)

Behavioral tests were conducted immediately after LIPUS. After that, rats were sacrificed, and their brains were removed for morphological and biochemical experiments. The flowchart of the experimental procedures is shown in Figure 1A.

### 2.3 Behavioral tests

The sucrose preference test (SPT) was performed at the beginning of the experiment and at the end of each week. The open field test (OFT) and forced swimming test (FST) were conducted at the end of the fourth week of LIPUS.

*SPT*: Rats were given access to two bottles of 1% sucrose solution for 24 hours followed by access to one bottle of 1% sucrose solution and one bottle of pure water for 24 hours. After water deprivation for the next 23 hours, rats were given preweighed bottles of 1% sucrose solution and pure water. Sucrose consumption and pure water consumption in the next hour were recorded. Total fluid consumption was the sum of sucrose consumption and pure water consumption. The SPI (%) was calculated as follow: sucrose consumption (g)/total fluid consumption (g) × 100%.

*OFT*: Each rat was placed in a gray polyvinyl chloride chamber (100 × 100 × 40 cm) for 5 minutes. It was carried out under the condition of weak light environment and the background noise intensity is constant at 60 dB. The test was performed during the dark phase of the light/dark cycle. The total distance (cm) traveled during this period was automatically measured using the SuperMaze behavior analysis system (Shanghai Xinruan Information Technology, Shanghai, China).

*FST*: A transparent cylinder (diameter = 30 cm) was filled with water at a depth of 37 cm and a temperature of 24 ± 1°C. Rats were subjected to 15‐min forced swimming training, preceding a 5‐min testing session 24 hours later. Immobility was defined as floating in the water, with only minor movements required to keep the head above the water. Ethovision XT software (Noldus, Netherlands) was used to analyze the duration of immobility during the 5‐min test period.

### 2.4 Surgery

Preoperatively, rats were anesthetized deeply with 1% pentobarbital sodium (0.5 ml/100 g, 50 mg/kg ip) and received a subcutaneous injection of meloxicam (2 mg/kg). During the surgical procedures, lidocaine (5 mg/kg, intradermal) was used for local anesthesia at the incision site and erythromycin ointment was used to smear animal eyes to prevent them from drying. A custom‐designed acoustic collimator was fixed on the skull covering the vmPFC (The center of the circle is bregma anteroposterior + 3.0 mm; medial‐lateral 0 mm) (Figure 1B) by screws (diameter 1 mm, length 2 mm) and dental resin (polymethyl methacrylate) using a stereotaxic apparatus (Stoelting, Wood Dale, IL, USA). Normal control rats had holes drilled into the skull, but the collimator was not affixed. The total duration of the surgical procedure was 15 minutes per rat. Then animals received a subcutaneous injection of Meloxicam (2 mg/kg) once daily for three days.

### 2.5 Pulsed ultrasound apparatus

**Figure 2:**
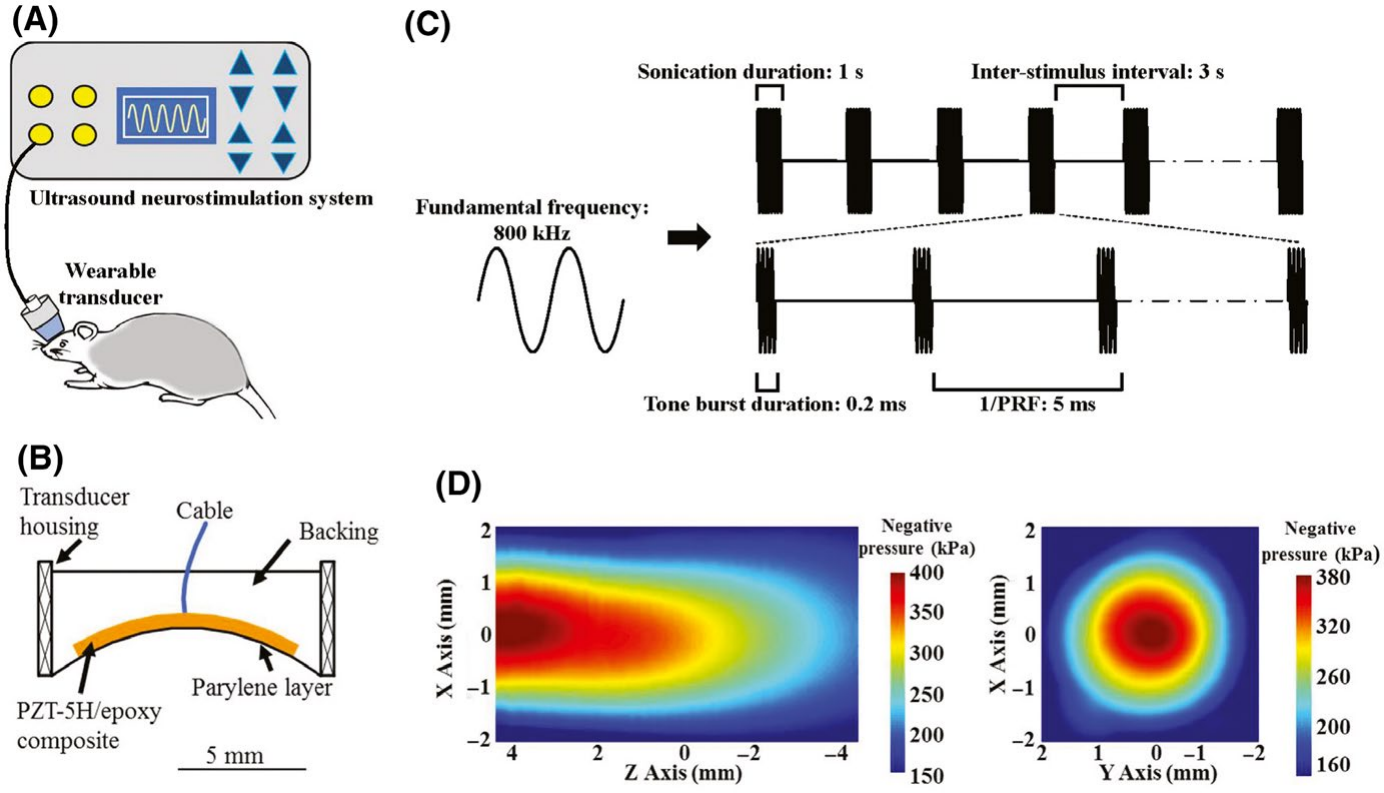
Illustration of ultrasound apparatus. A, Ultrasound neurostimulation system for freely moving rat. B, Schematic structure of the wearable transducer. C, Schematic of acoustic parameters. D, Acoustic intensity map in XY and XZ plane

A schematic diagram of the LIPUS stimulation system is shown in Figure 2A. LIPUS was generated by a home‐made wearable transducer (frequency: 800 kHz), mainly consist of 1‐3 PZT‐5H/epoxy composite, epoxy backing, and Parylene layer (Figure 2B), as described in Ref. 23. A PMMA (polymethyl methacrylate) collimator was fabricated to fix the transducer onto the target region of the rat skull. The total length of the collimator was approximately 9 mm from the face of the transducer to the end of the collimator, with an upper internal diameter of 14 mm. At 4 mm from the face of the transducer, there was a step in the collimator, and the diameter at the end of the collimator was 8 mm. During ultrasound stimulation, the transducer would be connected to the collimator. The collimator was filled with deionized, degassed water, to ensure successful delivery of ultrasound energy into the brain. The transducer and collimator weighed approximately 4 g in total, enabling rats to move freely while wearing it. The transducer was driven by a portable ultrasound system, which incorporated a waveform generator and a power amplifier.^31^ An impedance matching circuit was connected between the ultrasound system and transducer to improve energy conversion efficiency.

**Figure 3:**
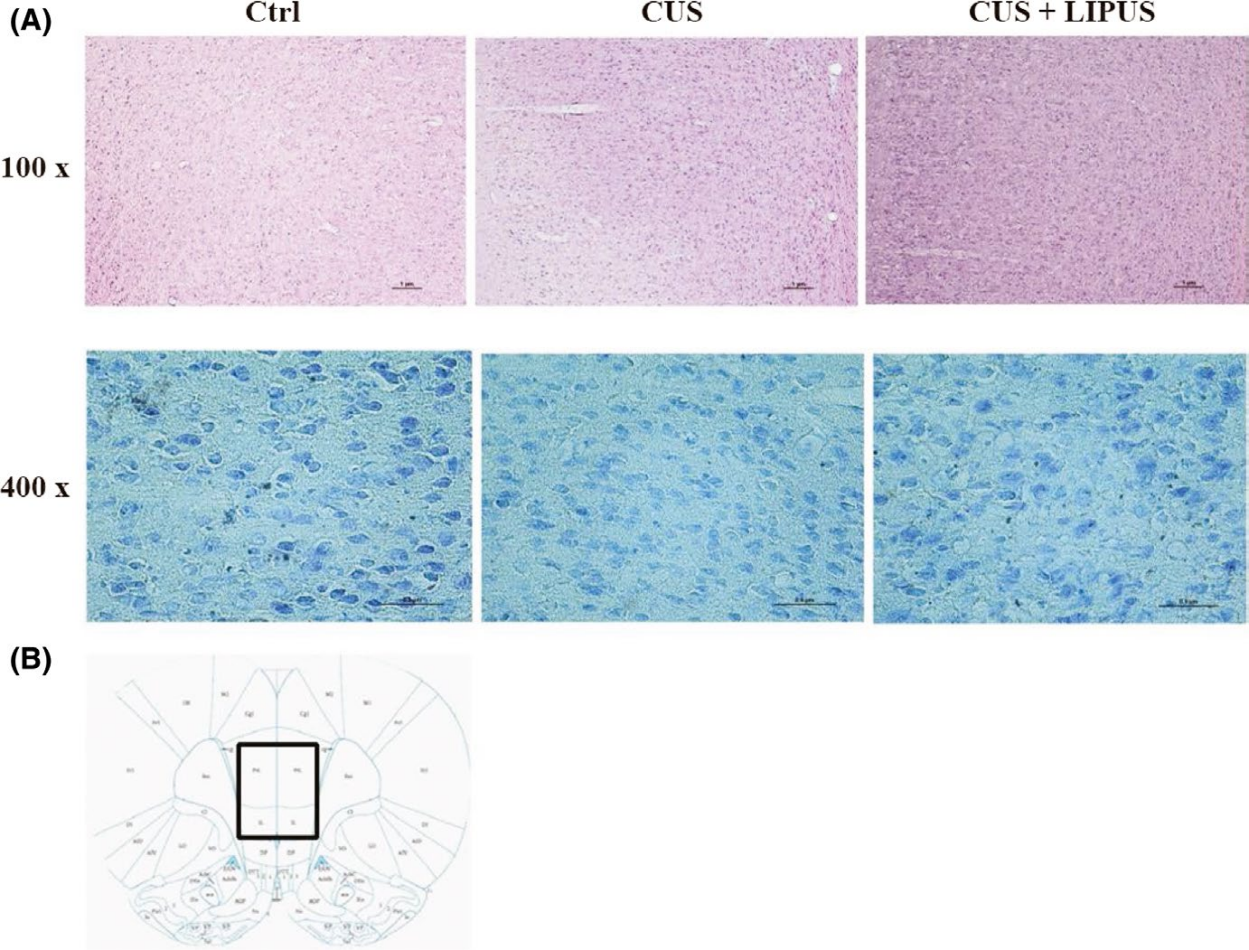
LIPUS does not cause gross tissue damage. A, Examples of HE staining (upper lane, scale bar = 1 μm) and Nissl staining (lower lane, scale bar = 0.5 μm) of the LIPUS stimulation site. Both staining methods revealed intact brain structure and neurons, with no signs of microhemorrhage, edema, cell necrosis, or local inflammatory responses in all groups of rats (n = 3 per group). B, Schematic illustration of the staining site

The LIPUS parameters applied to chronic stimuli (Figure 2B) were as follows: 200 Hz pulse repetition frequency (PRF), 4% duty cycle (DC), 0.2 ms tone burst duration (TBD), 1 seconds sonication duration (SD), and 3 seconds inter‐stimulus interval (ISI). The acoustic intensity field was measured in a degassed water tank by a calibrated hydrophone (SN2010, Precision acoustics, Dorchester, UK) equipped with a 3D ultrasound intensity measurement system (UMS3, Precision acoustics, Dorchester, UK). For XZ and XY scanning, the hydrophone was placed 1 mm away from the collimator. As shown in Figure 2C, the negative acoustic pressure was 0.40 MPa and the spatial‐peak temporal‐average intensity (I_spta_) was 248 mW/cm^2^, with a corresponding spatial‐peak pulse‐average intensity (I_sppa_) of 6.2 W/cm^2^ in free space. Figure 2D showed the acoustic intensity map in XY and XZ plane. The output acoustic distribution indicated that the full‐width‐at‐half‐maximum focal spot was 3.6 × 6.0 mm. Figure 1C showed the anatomical schematic of the stimulation area. After passing through the rat skull, the acoustic pressure decreased about 30%, with the negative acoustic pressure being 0.28 MPa and I_spta_ was 154 mW/cm^2^ (corresponding to I_sppa_ 3.84 W/cm^2^). To evaluate the safety of LIPUS, the temperature elevation induced by LIPUS stimulation was measured by an infrared thermal imager (R300, NEC Avio, Tokyo, Japan), to access heat accumulation in brain tissue. In brief, a rat head was transected, and the PFC and skull were preserved. The temperature of the tissue was monitored under LIPUS stimulation. The theoretical calculations of elevated temperature were less than 0.4°C in our study. We also examined the vmPFC using HE and Nissl staining to look for evidence of tissue damage. Both staining methods revealed intact brain structure and neurons, with no signs of microhemorrhage, edema, cell necrosis or local inflammatory responses in all groups of rats (Figure 3).

### 2.6 Immunohistochemistry and histological examinations

The c‐Fos expression in the vmPFC was detected using immunohistochemistry. One hour after LIPUS stimulation, rats were deeply anesthetized and then sacrificed by transcardial perfusion with saline and 4% paraformaldehyde respectively. The brain was removed and post‐fixed in 4% paraformaldehyde overnight at 4°C, followed by gradient dehydration with 20%, 30% sucrose until the brain completely sank to the bottom. Then, the brain was serially sectioned (35 μm thick) using a cryostat microtome. Two‐step immunohistochemistry was used to detect c‐Fos protein (mouse anti‐c‐Fos, 1:500, Abcam). The slices were then photographed with a Nikon light microscope. The c‐Fos‐positive cells were counted one by one in each small slice by the observers who were blind to the treatment condition. All c‐Fos‐positive cells in vmPFC were added up. The number of c‐Fos‐positive cells in six mice were obtained (n = 3 rats per group).

For histological examinations, sections were stained with standard hematoxylin‐eosin (HE) and Nissl staining and photographed with a Nikon light microscope (n = 3 rats per group).

### 2.7 Western blot

The prefrontal cortex was dissected and homogenized in RIPA lysis buffer and centrifuged. Western blot was performed using a 4%‐15% precast polyacrylamide gel. The gel was run at 100 V for 80 minutes before being transferred onto a nitrocellulose membrane at 2.5 A, 25 V for 3 minutes using the Trans‐Blot Turbo Transfer System (Bio‐Rad Laboratories, CA, USA). The membrane was then blocked for one hour using 5% skimmed milk in phosphate buffer saline at room temperature before being incubated with primary antibodies overnight at 4°C. Antibodies used in our study were as follows: BDNF antibody (1:1000, Abcam), β‐actin antibody (1:2000, Santa Cruz), tyrosine receptor kinase B (TrkB) antibody (1:400, BD Biosciences), phospho‐TrkB (pTyr706) antibody (1:500, Novus Biologicals), extracellular signal‐regulated kinase (ERK) antibody (1:1000, Cell Signaling), phospho‐ERK (Thr202/Tyr204) antibody (1:1000, Cell Signaling), protein kinase B (PKB/Akt) antibody (1:1000, Cell Signaling), phospho‐Akt (Ser473) antibody (1:1000, Cell Signaling), mTOR antibody (1:1000, Cell Signaling), phospho‐mTOR (Ser2448) antibody (1:1000, Cell Signaling), ribosomal protein S6 kinase (S6K) antibody (1:500, Cell Signaling) and phospho‐S6K (Thr389) antibody (1:500, Cell Signaling). Bands were detected and analyzed using a ChemiDoc™ Touch Imaging System (BIO‐RAD, CA, USA) (n = 4 rats per group). Levels of the BDNF, phosphorylated and activated forms of mTORC1, ERK, Akt, and S6K were determined by Western blot analysis. The β‐actin and total mTOR, ERK, Akt, and S6K were determined to control for loading differences. Full unedited gels were shown in Supplementary File.

### 2.8 Statistical analysis

Statistical analysis was performed using GraphPad prism 7 software. Data were presented as mean ± SEM. All data were firstly treated with normal distribution test. Data accorded with normal distribution and homogeneity of variance were compared by unpaired t test and one‐way ANOVA, or by nonparametric test. When there were 3 samples in each group, nonparametric (Mann‐Whitney) test was employed. The SPI and weight in CUS procedures and LIPUS were analyzed with two‐way repeated measures ANOVA. *P* values < 0.05 were considered statistically significant.

## 3 RESULTS

### 3.1 200 Hz LIPUS improves depression‐like behavior and increases neuronal activity in the vmPFC

**Figure 4:**
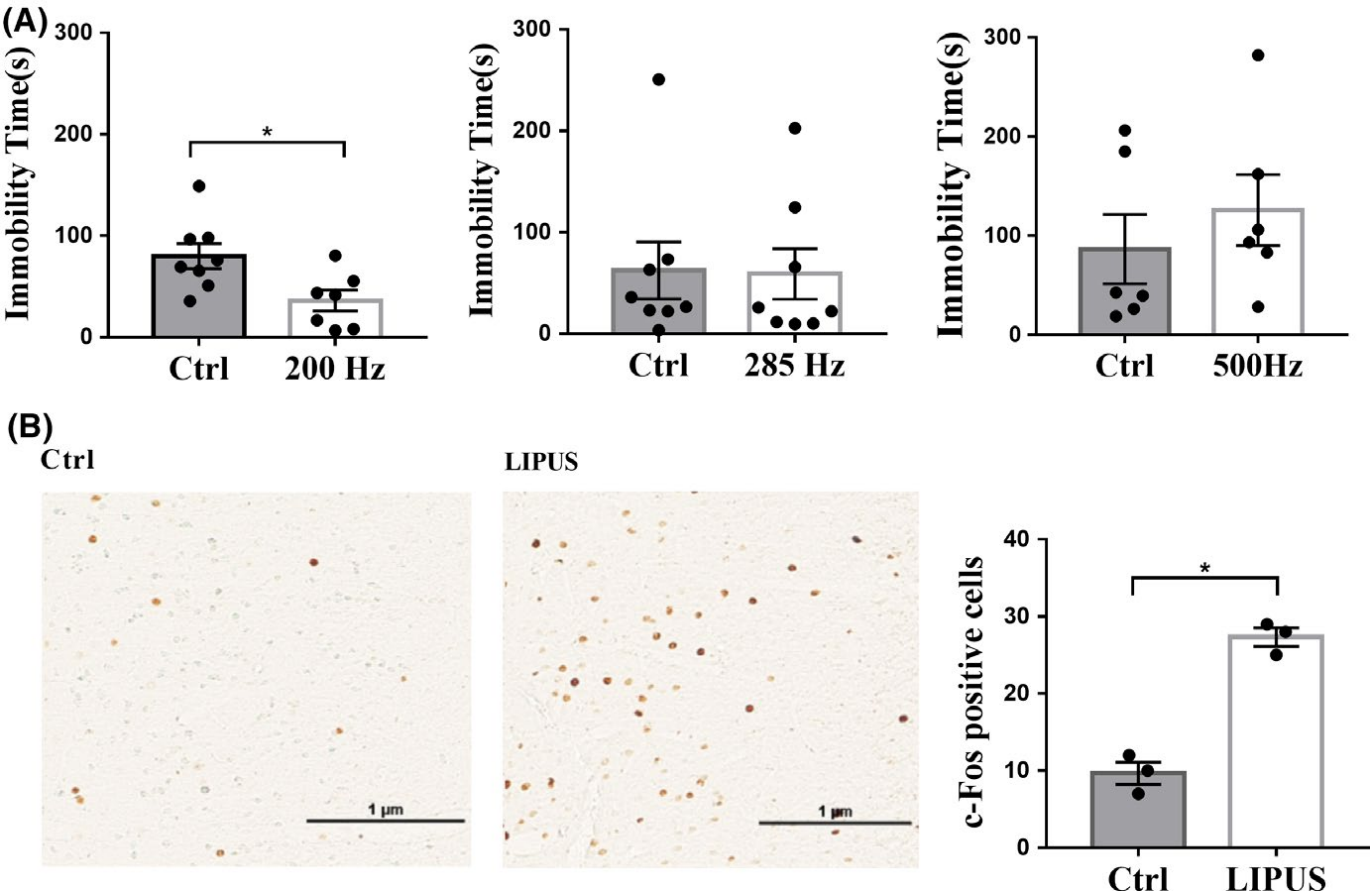
Screening of suitable stimulation parameters in normal rats. A, 200 Hz single LIPUS significantly decreased immobility time in the FST, while 285 Hz and 500 Hz LIPUS have no significant effects on immobility time (n = 6‐8 per group). B, The influence of single LIPUS stimulation on c‐Fos immunoreactivity in the vmPFC was determined 1 hour later. The number of c‐Fos‐positive cells in the vmPFC was significantly increased in the LIPUS group compared with control group (n = 3 per group). Data are expressed as mean ± SEM. *P < 0.05, vs control group

In order to obtain suitable stimulation parameters, we first examined the behavioral effects of 30‐min single LIPUS stimulation of the vmPFC with three different PRFs (200 Hz, 285 Hz and 500 Hz) in nonstressed rats. After 24 hours, we found that only 200 Hz LIPUS could significantly decrease immobility time in the FST (Figure 4A; t = 2.691 *P* < 0.05). Next, we examined the influence of LIPUS on levels of c‐Fos immunoreactivity. As shown in Figure 4B, single LIPUS stimulation significantly increased the number of c‐Fos‐positive cells in the vmPFC compared with sham‐LIPUS (Z = −1.964, *P* < 0.05). Taken together, these results suggest that single 200 Hz LIPUS of the vmPFC can produce antidepressant‐like behavioral effects, and rapidly enhance neuronal activity.

### 3.2 LIPUS improves depression‐like behaviors in CUS rats

**Figure 5:**
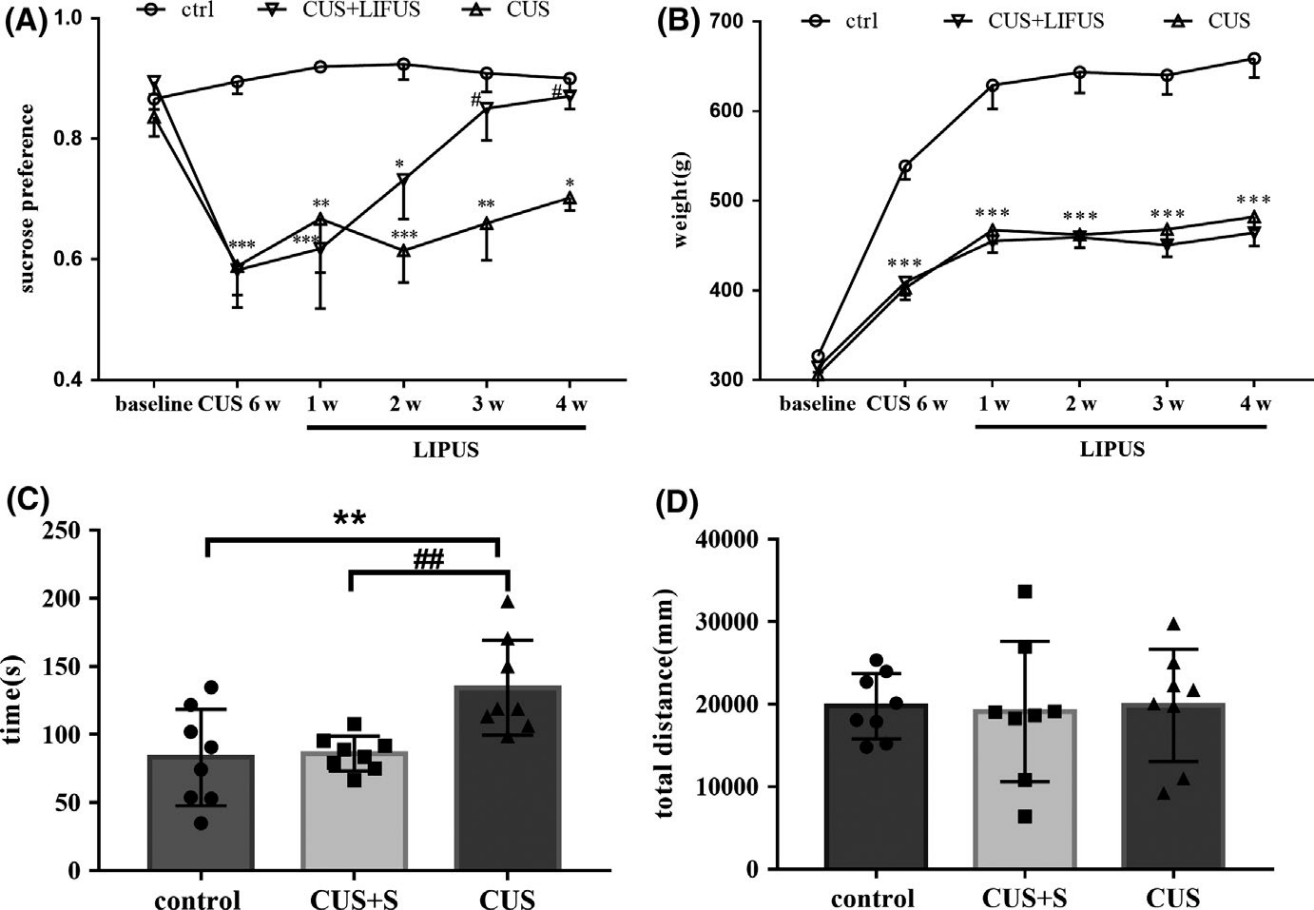
Four weeks of LIPUS stimulation of vmPFC can improve depression‐like behaviors in CUS rats. A, Changes in body weight. B, Six weeks of CUS greatly decreased the SPI, and LIPUS stimulation significantly increased the SPI from the third week. C, Four weeks of LIPUS stimulation reversed the increased immobility time in the FST in CUS rats. D, No statistical difference was detected in the total distance in the OFT among the three groups. Data are expressed as mean ± SEM (n = 8 per group). *P < 0.05, **P < 0.01 and ***P < 0.001 vs control group; *^#^P* < 0.05, *^##^P* < 0.01 and *^###^P* < 0.001 vs CUS group

To further examine the antidepressant effects of chronic LIPUS stimulation, we established a CUS rat model of depression. Significantly lower body weights were observed in CUS rats than control rats at each time point after CUS, and LIPUS had no effect on the body weight of CUS rats (Figure 5A; all *P* < 0.001). In the SPT, six weeks of CUS induced a significant reduction in SPI in model rats compared with control rats (*P* < 0.001). LIPUS significantly increased sucrose preference in CUS rats beginning in the third week, and this increase was continued in the fourth week (Figure 5B; 3rd week, *P* < 0.05; 4th week, *P* < 0.05). LIPUS significantly decreased immobility time in the FST among CUS rats (Figure 5C; *P* < 0.01). In addition, there was no significant difference in movement distance among the three groups in the OFT (Figure 5D). Taken together, these results suggest that chronic LIPUS of the vmPFC has significant antidepressant‐like effects, as shown by improving measures of anhedonia and behavioral despair in a rat model of depression.

### 3.3 LIPUS enhances BDNF‐mTORC1 signaling pathways in the PFC

**Figure 6:**
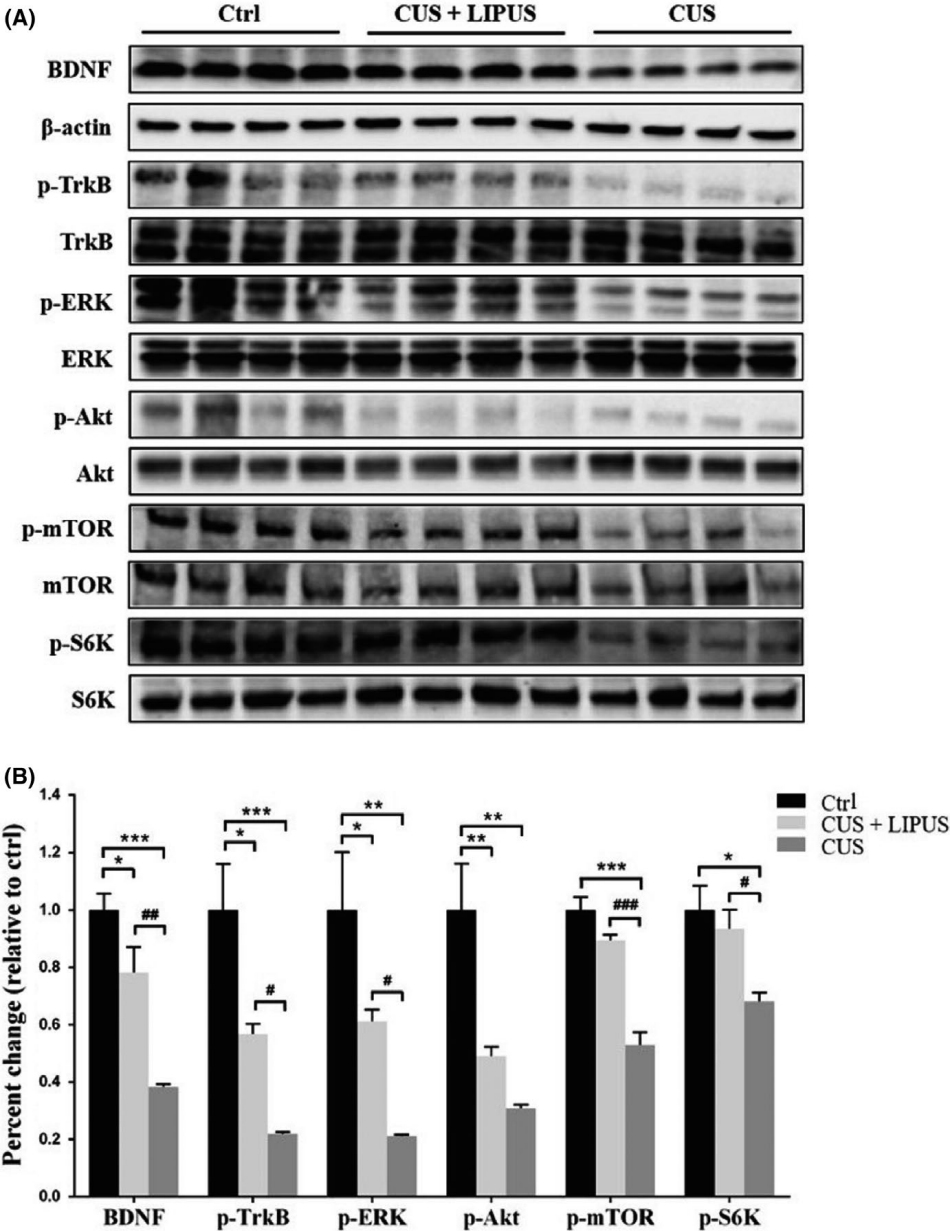
Four weeks of LIPUS stimulation enhances BDNF‐mTORC1 signaling pathway in the PFC of CUS rats. Levels of BDNF, phosphorylated forms of TrkB, ERK, Akt, mTOR, and S6K were measured by Western blot analysis. Levels of β‐actin and total corresponding nonphosphorylated proteins were used as loading controls, respectively. A, Representative immunoblot images. B, Results are expressed as fold change relative to control group. Data are expressed as mean ± SEM (n = 4 per group). *P < 0.05, **P < 0.01 and ***P < 0.001 vs control group; *^#^P* < 0.05, *^##^P* < 0.01 and *^###^P* < 0.001 vs CUS group

Next, we examined whether the mTROC1 signaling pathway in the PFC was involved in the antidepressant‐like behavioral effects of LIPUS (Figure 6). Western blot analysis revealed that phospho‐mTORC1 levels were significantly decreased in CUS rats compared with those in control rats (*P* < 0.001), and this reduction was partially reversed by four weeks of treatment with LIPUS (*P* < 0.001). Similar results were obtained for the levels of phospho‐S6K (*P* < 0.05), one of the main downstream effectors of mTORC1.

The mitogen‐activated protein kinase (MAPK)/ERK and phosphatidyl inositol‐3 kinase (PI3K)/Akt pathways can activate mTORC1 signaling.^40^ We found that both ERK and Akt activities were dramatically decreased in CUS rats (both *P* < 0.01). LIPUS treatment restored the activity of ERK (*P* < 0.05), but not Akt. BDNF/TrkB signaling is able to transduce both MAPK/ERK and PI3K/Akt to activate mTORC1.^33^ We also found that LIPUS partially restored both mature BDNF (*P* < 0.01) and phospho‐TrkB levels (*P* < 0.05) that were reduced by CUS, though not to the level of the control group. These results indicate that LIPUS may enhance BDNF/ERK/mTORC1 signaling pathways after suppression by CUS.

## 4 DISCUSSION

Noninvasive neuromodulation treatment for neuropsychiatric disorders with LIPUS is still in the preclinical stage and further investigations are needed to establish the potential for clinical efficacy in treating depression. To our knowledge, the present study is the first to demonstrate the antidepressant‐like effects of LIPUS in awake and freely moving rats using a classical depression model. We found that chronic stimulation of the vmPFC with LIPUS significantly improved depression‐like behaviors in CUS rats with no histological evidence of damage to the brain. Moreover, LIPUS was able to partially reverse CUS‐induced downregulation of BDNF/ERK/mTORC1 signaling pathways in the PFC.

The stimulation site and parameters are two important factors affecting the efficacy of LIPUS. We chose to target the vmPFC (homologous to the human subcallosal cingulate gyrus) because there is substantial evidence that it is a key node of cortical and subcortical networks involved in a variety of social, cognitive, and affective functions.^41^ In depression, dysfunction in vmPFC activity is associated with abnormal regulation of negative emotion.^42^ DBS of the subcallosal cingulate gyrus has been demonstrated to be effective in treating treatment‐resistant depression in a number of clinical studies.^43^, ^44^, ^45^, ^46^, ^47^ In addition, excitation of the vmPFC by transcranial direct current stimulation (tDCS) can enhance perceptual processing of pleasant relative to unpleasant stimuli in human.^48^ In combination with data from the literature, our findings support the notion that LIPUS targeting the vmPFC could be a promising treatment for depression.

Ultrasound waves at frequencies in the 0.5‐1.0 MHz range can penetrate the skull^49^, ^50^, ^51^ to achieve clinically relevant biological effects.^52^ Here, we selected 800 kHz as the fundamental frequency to ensure transcranial effects with relatively precise spatial targeting. The biological effects of ultrasound include cavitation, thermal induction, and mechanical stimulation. To induce cavitation in brain tissue lacking microbubbles, the ultrasound pressure needs to be over 40 MPa.^53^ In our study, the negative peak pressure (0.28 MPa) was far below the threshold for cavitation, so this is unlikely to be the mechanism underlying our observed effects. In addition, a low duty cycle of 4% was used to avoid thermal ultrasound effects. The temperature elevation measured by the thermal infrared imager was less than 0.4°C. Rinaldi et al reported that a 0.75°C temperature elevation induced by ultrasound stimulation had minimal biological effects on in vitro hippocampal slices.^54^ As expected, we did not observe any tissue damage or hemorrhage along the LIPUS sonication path, based on our histological analyses. The theoretical thresholds for ultrasound tissue damage are thus consistent with our experimental results, both of which suggest that LIPUS with our study parameters is relatively safe. The US Food and Drug Administration specifies that safe ultrasound imaging should have a maximum mechanical index (MI) = 1.9, Ispta = 720 mW/cm^2^, and Isppa = 190 W/cm^2^. Our ultrasound parameters (MI = 0.28, Ispta = 154 mW/cm^2^ Isppa = 3.84 W/cm^2^) were far below the FDA limits, providing further assurance that the tested parameters are safe and unlikely to damage the brain.

Although there is extensive research on the neuromodulatory effects of LIPUS, the specific underlying mechanisms remain poorly understood. Nevertheless, it is clear that LIPUS stimulation can directly activate hippocampal neurons in vitro.^16^, ^55^ Previous studies also suggest that mechanical ultrasound effects can modulate the firing patterns of primary cultures of hippocampal cells, and increase excitatory postsynaptic current (EPSCs) frequency in rat hippocampal pyramidal neurons.^16^, ^56^ Previous studies have demonstrated that LIPUS produces excitatory or inhibitory neuromodulation largely depending on its parameters,^57^ but there is still no consensus on what parameters of LIPUS stimulation can produce excitatory or inhibitory effects. Here, we demonstrated that LIPUS stimulation with the parameters chosen in our study can activate vmPFC neurons in vivo. Consistently, several previous studies also reported neuroexcitatory effects of LIPUS with similar parameters.^9^, ^12^, ^58^, ^59^ The parameters that proposed to have antidepressant effect are quite different [5, 6]. The parameter used in our research was unique, which may give a reference to future research. Further work is needed to clarify which type of neuron that play critical roles in the effect of ultrasound.

We also investigated the possibility that mTORC1 signaling could be involved in the molecular mechanisms underlying the antidepressant‐like effects of LIPUS to carry on a deeper research to the mechanism of ultrasound neuromodulation. Activation of the mTORC1 pathway is critical for many nervous system functions, including neuronal excitability. The upstream activators of mTORC1 signaling are Akt and ERK, which inhibit tuberous sclerosis complexes (TSC1 and TSC2), allowing activation of mTORC1.^60^ The downstream effectors of mTORC1 are the S6K and the eukaryotic initiation factor 4E (eIF4E)‐binding proteins (4E‐BP), both of which regulate protein synthesis.^61^ Human postmortem and animal studies have demonstrated impaired mTORC1 signaling in the PFC in depression.^62^ Moreover, mTORC1 signaling plays a critical role in the efficacy of classical antidepressants,^35^, ^63^ as well as rapid‐acting antidepressant agents, such as ketamine^64^, ^65^ and scopolamine.^34^ Similarly, antidepressant effects of neuromodulatory electroconvulsive therapy (ECT) and DBS are also reported to be mediated by the mTOR pathway.^66^, ^67^ Here, we found reduced phosphorylation levels of Akt, ERK, mTORC1 and S6K in CUS rats. Chronic LIPUS treatment partially restored the levels of all of these molecules except Akt.

We also investigated potential upstream regulators of ERK. BDNF is a critical neurotrophic factor that has been associated with the pathophysiology of depression. Through binding to its high‐affinity receptor TrkB, BDNF can lead to diverse physiological effects by regulating a complex cascade of post‐receptor pathways, involving MAPK/ERK, PI3K/Akt or phospholipase C‐γ (PLC‐γ).^68^ Previous studies suggest that LIPUS can enhance endogenous BDNF expression in cerebral cortex astrocytes in vivo and in vitro through activation of TrkB/Akt and calcium/CaMK signaling pathways.^52^, ^69^ Consistent with these observations, our results showed that LIPUS reversed the stress‐induced reduction in BDNF expression and TrkB phosphorylation.

However, there were some differences in our data compared to these other studies when examining downstream signaling molecules. We found that LIPUS significantly rescued pERK levels, but the effect on pAkt, though in the same direction as pERK, was not statistically significant. These differences could arise from the use of very different model systems (animal versus cell) and the duration of LIPUS treatment (chronic versus acute). Taken together, our data suggest that chronic LIPUS stimulation may activate ERK/mTORC1 signaling in the PFC through BDNF/TrkB. Although our results are not enough to clarify the mechanism, they can provide molecular evidence supporting the improvement of depression by LIPUS.

Meanwhile, our research had some practice value in future clinical use of LIPUS. First, we used a portable ultrasound system that has been developed independently. This portable ultrasound system is much more convenient for researchers to achieve ultrasonic neurostimulation, and we applied LIPUS to awake and free‐moving animals rather than anesthetized ones. This way seems more suitable for future application in clinic. Second, the CUS depression model has been proved to be better in construct validity and face validity compared with other depression animal models.^70^ In addition, this research made a preliminary exploration of the safety of LIPUS.

Finally, several limitations in the present study should be addressed. First, we only observed the effects of LIPUS stimulation of the vmPFC. Further studies will be needed to explore the role of other depression‐related brain regions, such as lateral habenula and nucleus accumbens. Second, although we found behavior and molecular effects of LIPUS, the excitatory or inhibitory neuromodulation changes following LIPUS are expected to be clarified by in vivo electrophysiological recording. Third, sonication parameters can affect the biological effects of LIPUS. Therefore, further work that addresses these limitations is needed to strengthen the findings of our study.

## 5 CONCLUSIONS

In conclusion, this study demonstrated that four weeks of LIPUS stimulation of the vmPFC can improve depression‐like behaviors in a CUS model in rats. Potential mechanisms underlying this behavioral effect could be related to enhancement of BDNF/ERK/mTORC1 signaling in the PFC. To our knowledge, this is the first study to explore the antidepressant effects and associated mechanisms of chronic LIPUS stimulation on a classic depression model. Although further work is of course required, LIPUS could be regarded as a promising, noninvasive neuromodulation tool for the clinical treatment of depression.

## CONFLICT OF INTEREST

The authors declare no conflict of interests.

## Supporting information

Supplementary Material

## Data Availability

The data that support the findings of this study are available from the corresponding author upon reasonable request.
