# Supplementary figures and images for: Low‐intensity pulsed ultrasound ameliorates depression‐like behaviors in a rat model of chronic unpredictable stress
Source: CNS Neurosci Ther. 2020 Oct 28;27(2):233–43. doi: 10.1111/cns.13463 (PMC7816209; doi:10.1111/cns.13463)

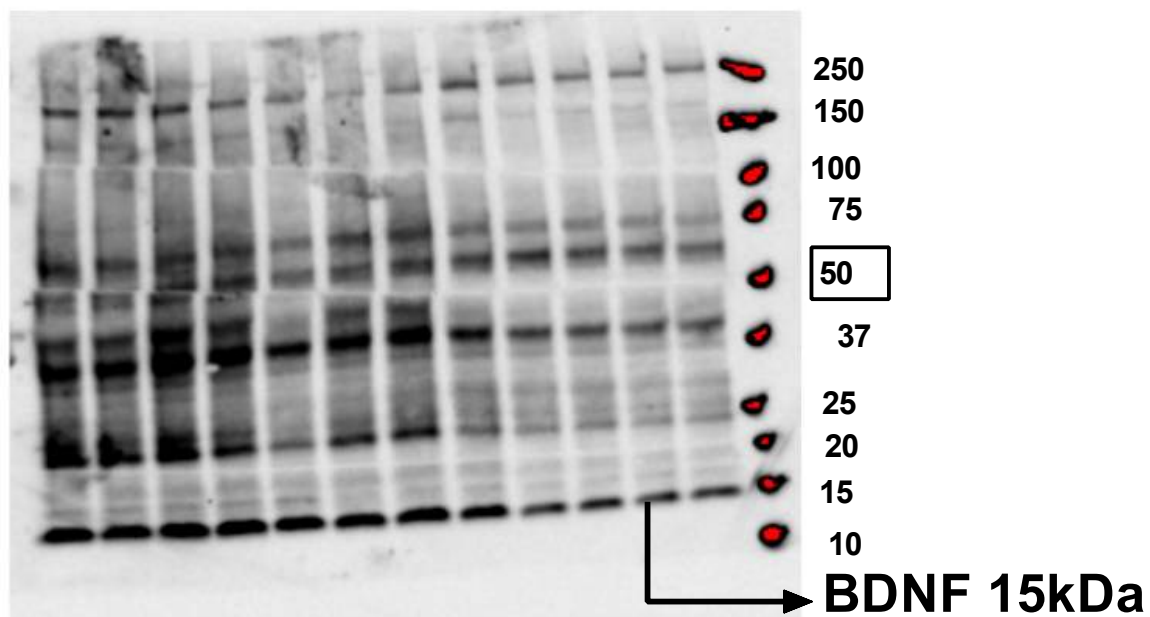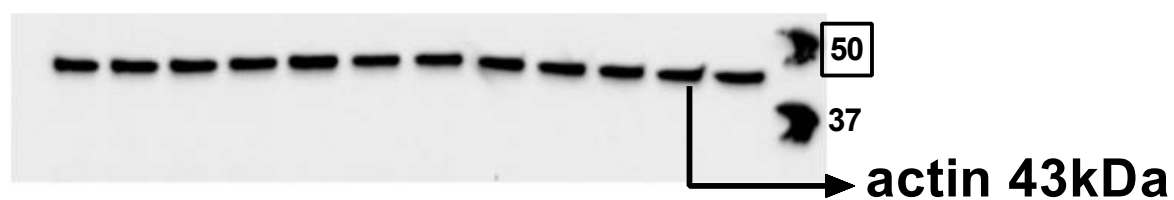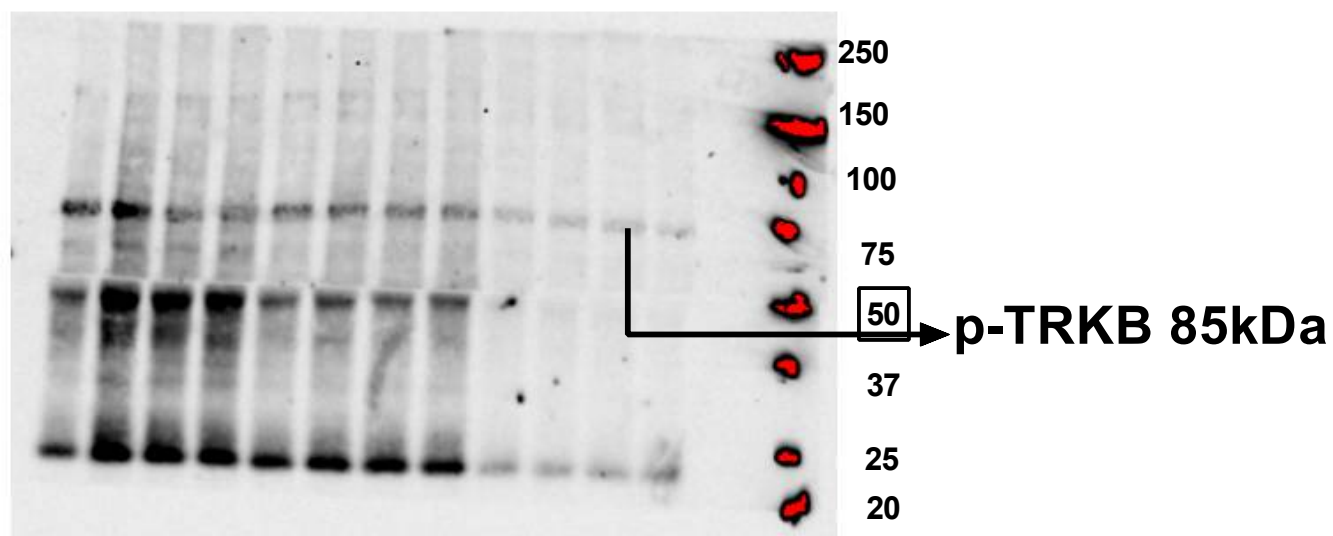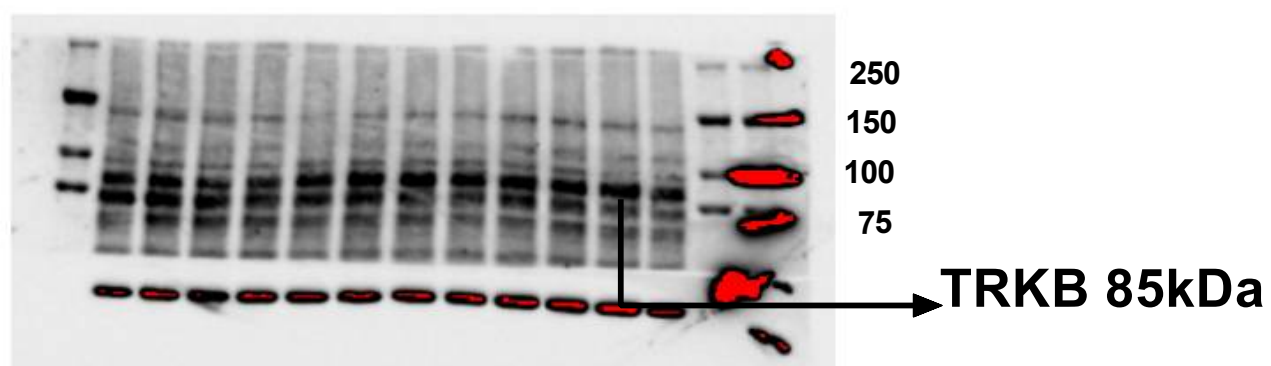

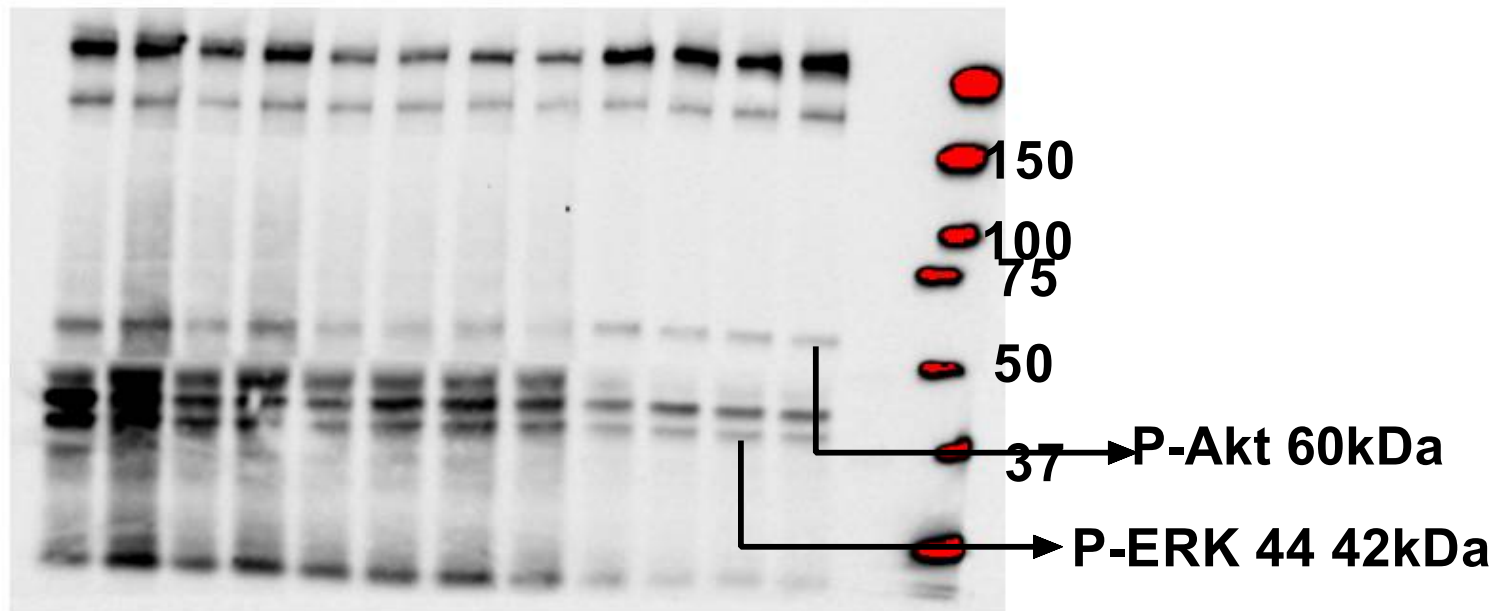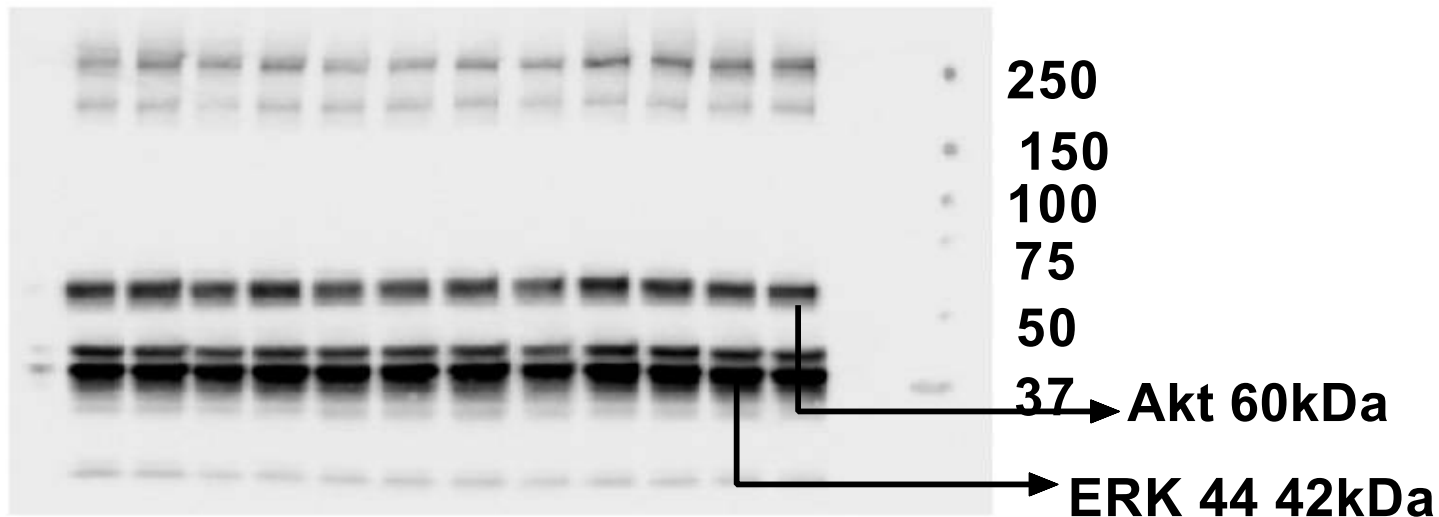

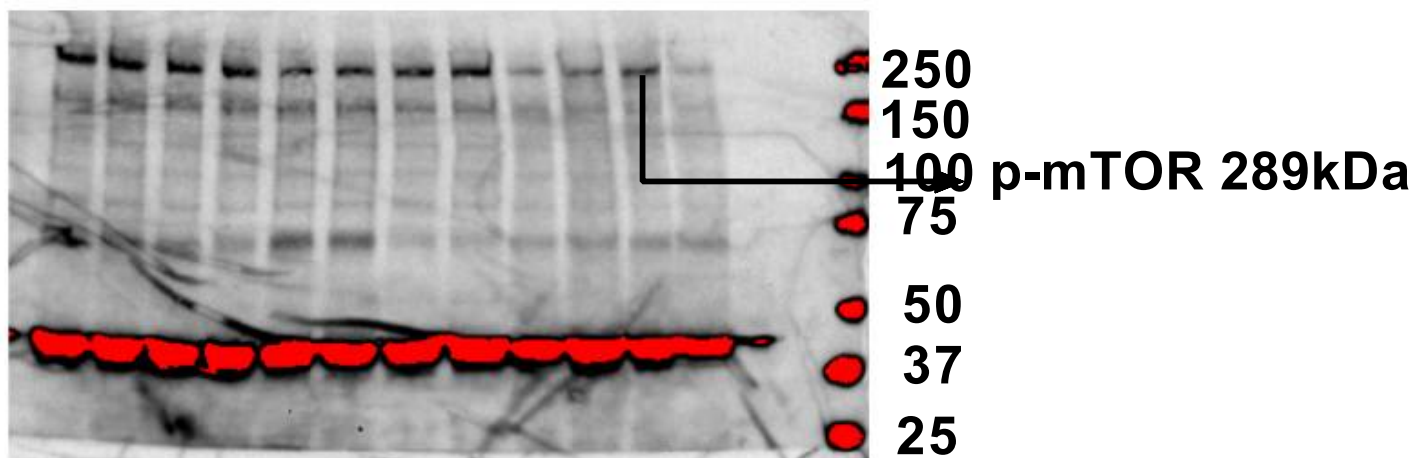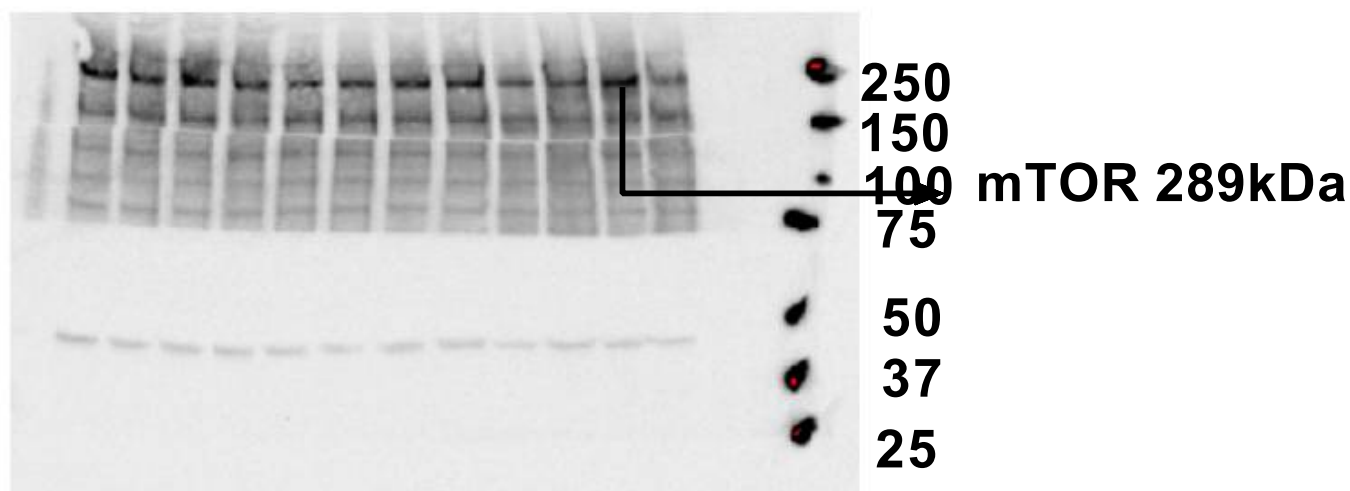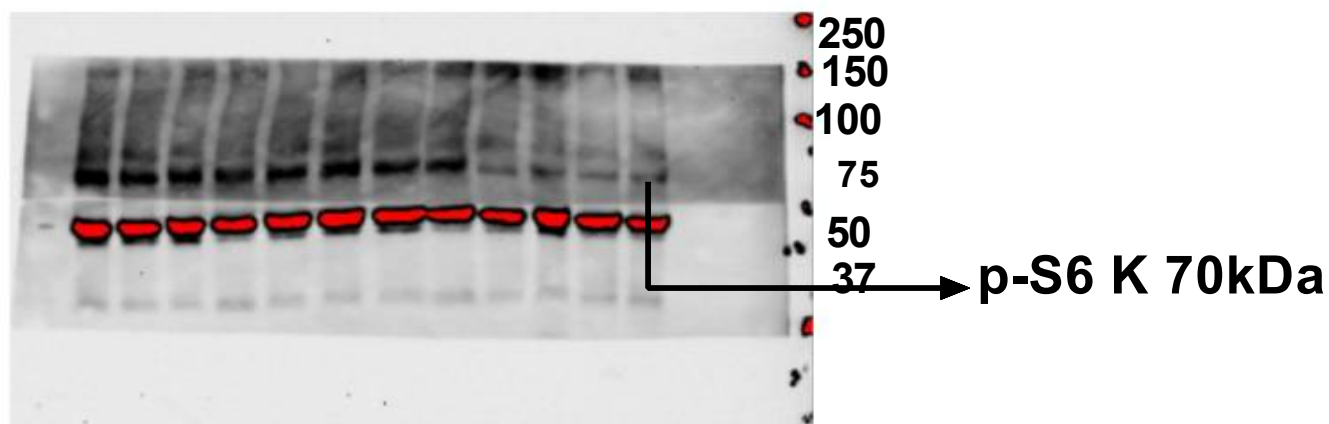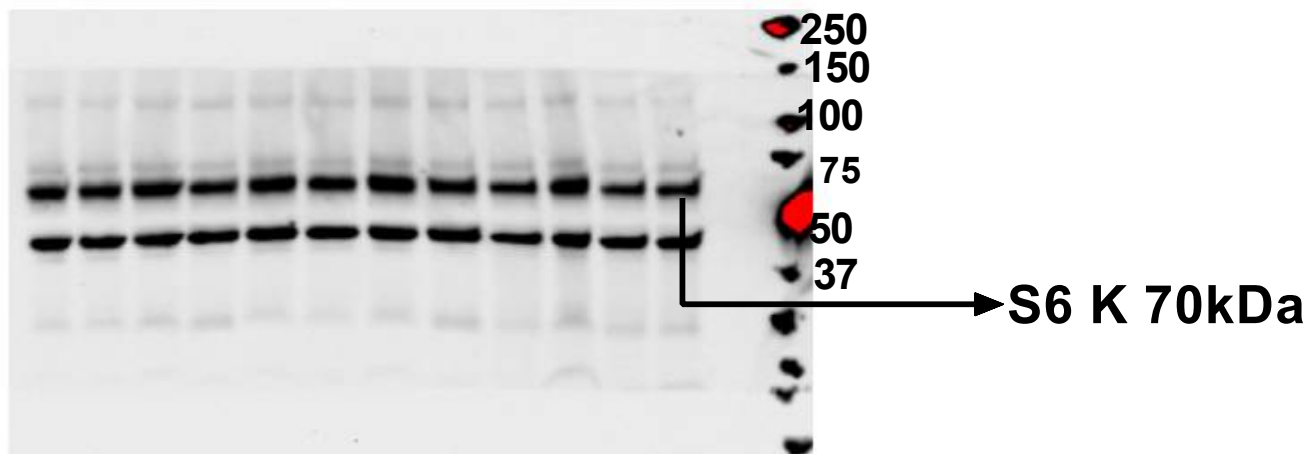

Supplement: Supplementary file 1 — Supplementary Material [file CNS-27-233-s001.pdf]
